# Interleukin 1 alpha administration is neuroprotective and neuro-restorative following experimental ischemic stroke

**DOI:** 10.1186/s12974-019-1599-9

**Published:** 2019-11-14

**Authors:** Kathleen E. Salmeron, Michael E. Maniskas, Danielle N. Edwards, Raymond Wong, Ivana Rajkovic, Amanda Trout, Abir A. Rahman, Samantha Hamilton, Justin F. Fraser, Emmanuel Pinteaux, Gregory J. Bix

**Affiliations:** 1 Sanders Brown Center on Aging, University of Kentucky, Lexington, KY 40536 USA; 2 Department of Neuroscience, University of Kentucky, Lexington, KY 40536 USA; 3 Department of Neurosurgery, University of Kentucky, Lexington, KY 40536 USA; 4 Department of Neurology, University of Texas Health Science Center, Houston, TX 77030 USA; 5 Faculty of Biology, Medicine and Health, A.V. Hill Building, University of Manchester, Oxford Road, Manchester, M13 9PT UK; 6 Department of Neurology, University of Kentucky, Lexington, KY 40536 USA; 7 Center for Advanced Translational Stroke Science, University of Kentucky, Lexington, KY 40536 USA

**Keywords:** Interleukin 1 alpha, Stroke, Therapeutic, Neuroprotection, Neurorepair, Angiogenesis, Perlecan, LG3, Mouse model

## Abstract

**Background:**

Stroke remains a leading cause of death and disability worldwide despite recent treatment breakthroughs. A primary event in stroke pathogenesis is the development of a potent and deleterious local and peripheral inflammatory response regulated by the pro-inflammatory cytokine interleukin-1 (IL-1). While the role of IL-1β (main released isoform) has been well studied in stroke, the role of the IL-1α isoform remains largely unknown. With increasing utilization of intravenous tissue plasminogen activator (t-PA) or thrombectomy to pharmacologically or mechanically remove ischemic stroke causing blood clots, respectively, there is interest in pairing successful cerebrovascular recanalization with neurotherapeutic pharmacological interventions (Fraser et al., J Cereb Blood Flow Metab 37:3531–3543, 2017; Hill et al., Lancet Neurol 11:942–950, 2012; Amaro et al., Stroke 47:2874–2876, 2016).

**Methods:**

Transient stroke was induced in mice via one of two methods. One group of mice were subjected to tandem ipsilateral common carotid artery and middle cerebral artery occlusion, while another group underwent the filament-based middle cerebral artery occlusion. We have recently developed an animal model of intra-arterial (IA) drug administration after recanalization (Maniskas et al., J Neurosci Met 240:22–27, 2015). Sub groups of the mice were treated with either saline or Il-1α, wherein the drug was administered either acutely (immediately after surgery) or subacutely (on the third day after stroke). This was followed by behavioral and histological analyses.

**Results:**

We now show in the above-mentioned mouse stroke models (transient tandem ipsilateral common carotid artery (CCA) and middle cerebral artery occlusion (MCA) occlusion, MCA suture occlusion) that IL-1α is neuroprotective when acutely given either intravenously (IV) or IA at low sub-pathologic doses. Furthermore, while IV administration induces transient hemodynamic side effects without affecting systemic markers of inflammation, IA delivery further improves overall outcomes while eliminating these side effects. Additionally, we show that delayed/subacute IV IL-1α administration ameliorates functional deficit and promotes neurorepair.

**Conclusions:**

Taken together, our present study suggests for the first time that IL-1α could, unexpectedly, be an effective ischemic stroke therapy with a broad therapeutic window.

## Background

Ischemic stroke is a leading cause of death and long-term disability worldwide [28]. While the advent of intravenous (IV) t-PA and endovascular mechanical thrombectomy to recanalize intracranial vessel occlusions has had a major impact on outcome, most patients are left with some significant disability, underscoring the need for new pharmacotherapies to improve stroke recovery [3, 14, 18, 34]. Inflammation is a key contributor to brain injury following stroke; as such, it has great potential as an area for therapeutic intervention [11, 34]. Generally, post-stroke inflammation is characterized by the expression of inflammatory mediators via activated immune cells within the core of the infarct (in the brain parenchyma), such as microglia and astrocytes [36, 40, 48]. This leads to an activation of surrounding cerebrovasculature, and a subsequent opening of the blood-brain barrier (BBB) resulting in edema and widespread secondary damage by peripheral immune cells [40]. More recently, this invasion of peripheral cells has been shown to contribute to long-term neuroinflammation, as well as to post-stroke cognitive decline [13]. While many preclinical and clinical trials have examined the use of anti-inflammatory therapeutics [46], attempts at targeting post-stroke inflammation have failed to significantly improve patient prognosis [12, 15].

The role of inflammation driven by the pro-inflammatory cytokine interleukin (IL)-1 during post-stroke injury has been the focus of intense research [16, 33]. Indeed, preclinical studies have demonstrated the deleterious actions of interleukin-1 (IL-1) after stroke, while blocking its actions is beneficial in preclinical [35] and clinical [15] settings. The large majority of studies have focused on the role of IL-1beta (β) (main released isoform) and demonstrated that interleukin-1 beta isoform (IL-1β) is a primary mediator of central and peripheral inflammation after stroke [7]. Many preclinical studies have focused on modifying IL-1β levels by exogenous administration of recombinant IL-1β or selective anti-IL-1β neutralizing antibodies on experimental ischemia in rodent models [24]. However, the role of IL-1alpha (α) (main intracellular isoform) during post-stroke inflammation is largely unknown. Recent published works have demonstrated marked differences between mechanisms of expression and action of these two cytokines, suggesting that interleukin-1 alpha isoform (IL-1α) might exert specific actions; while IL-1α generally remains cytoplasmic, it can be released during cell death or by mechanisms that are different from that of IL-1β [7]. Previous studies have demonstrated differential actions of both cytokines in various paradigms of inflammation [8]. In stroke, brain IL-1α expression precedes that of IL-1β and occurs predominantly in microglia localized to focal neuronal and BBB injury in this acute period [23]. Furthermore, polymorphisms in the human *IL1A gene*, as opposed to the *IL1B* gene, result in higher incidence of vascular malformation, and/or higher risk of ischemic stroke [44, 45], further suggesting that IL-1α may exert different actions than IL-1β in ischemic stroke.

We have recently described the angiogenic effects of IL-1α in post-stroke angiogenesis in vitro [39]. The present study extends our previous findings and sought to test the hypothesis that acute or subacute exogenous intravenous (IV, given acutely or subacutely) or intra-arterial (IA, given acutely) administration of subpathological doses of IL-1α could have well-tolerated beneficial neuroprotective or neuroreparative effects, respectively, and if so, how it might exert these effects. This study may have vital implications by proposing for the first time that complete inhibition of post-stroke neuroinflammation may have detrimental effects, while sustaining low-grade chronic inflammation (i.e., therapeutic inflammation) might be used as new effective therapy for brain tissue repair and functional recovery after stroke.

## Methods

### Recombinant IL-1α protein preparation

Upon arrival, mouse recombinant IL-1α (R&D Systems, Minneapolis, MN, USA) was diluted in sterile phosphate-buffered saline containing 0.1% low endotoxin bovine serum albumin (BSA) (also used as vehicle control). To avoid freeze thaw cycles, the diluted stock solution (50 μg/mL) was then aliquoted and frozen for dilution to the desired dose on the day of surgery or treatment.

### Surgical methods

Experimental protocols were approved by the Institutional Animal Care and Use Committee of the University of Kentucky (USA), as well as the Home Office (United Kingdom, UK), and experiments were performed in accordance with the Guide for the Care and Use of Laboratory Animals of the National Institutes of Health as well as the ARRIVE guidelines.

#### Tandem ipsilateral common carotid and middle cerebral artery occlusion stroke model

Briefly, 3-month-old male C57BL/6 mice (Jackson Labs, Bar Harbor, Maine, USA) or perlecan hypomorph mice (expressing 10% of normal total perlecan levels, generated in a C57BL/J6 background, hereafter referred to as pln KO mice) were subjected to transient tandem ipsilateral common carotid artery (CCA)/middle cerebral artery (MCA) occlusion (MCAo) for 60 min [22], followed by reperfusion of both arteries for up to 7 days. A small burr hole was made in the skull to expose the MCA and a metal wire with a diameter of 0.005 in was placed under the artery. Slight elevation of the metal wire causes visible occlusion of the MCA. The CCA was then isolated and occluded using an aneurysm clip. Diminished blood flow was confirmed with laser Doppler perfusion monitor (Perimed, USA) positioned slightly distal to the burr hole, and only those animals with a diminished blood flow of at least 80% and re-establishment of at least 75% of baseline levels were included in subsequent experimentation. For studies involving vital statistics, heart rate, pulse distension, and core temperature was monitored using MouseOx Small Animal Pulse Oximeter (Starr Life Sciences Corp., Oakmont, PA, USA). Heart rate and pulse distension were monitored via thigh clamp while core temperature was monitored via rectal probe.

#### Middle cerebral artery occlusion (filament) model

In experiments involving delayed/subacute IL-1α administration, 3-month-old male C57BL/J6 mice underwent MCAo as previously described [47]. Briefly, a hole was made into the temporalis muscle (6 mm lateral and 2 mm posterior from bregma) to allow a 0.5-mm-diameter flexible laser Doppler probe to be fixed onto the skull and secured in place by tissue adhesive (Vetbond). A midline incision was made on the ventral surface of the neck and the right CCA isolated and ligated. Topical anesthetic (EMLA, 5% prilocaine and lidocaine, AstraZeneca, UK) was applied to skin incision sites prior to incision. The internal carotid artery (ICA) and the pterygopalatine artery were temporarily ligated. A 6-0 monofilament (Doccol, Sharon, MA, USA) was introduced into the ICA via an incision in the CCA. The filament was advanced approximately 10 mm distal to the carotid bifurcation, beyond the origin of the MCA. After 20 min of occlusion, the filament was withdrawn back into the CCA to allow reperfusion to take place. Relative cerebral blood flow (CBF) was monitored following MCAo, during which time relative CBF had to reduce by at least 70% of pre-ischemic values for inclusion. The wound was sutured, and mice received a subcutaneous bolus dose of saline for hydration (500 μL) and a general analgesic (Buprenorphine, 0.05 mg/kg injected subcutaneously). Animals were kept at 37 °C during surgery and then at 26–28 °C (room temperature) while they recovered from anesthesia and surgery, before being transferred back to ventilated cages suspended over a heating pad for 24 h post-surgery with free access to mashed food and water in normal housing conditions.

#### Intra-arterial drug administration

Animals in the IA drug delivery cohort underwent IA drug delivery as previously described [25]. Briefly, the mouse was placed in a supine position with the previously isolated CCA exposed. Following the CCA superiorly to its bifurcation point, the ICA and external carotid artery (ECA) respectively) were identified and three lengths of 6-0 suture were placed under the ECA, ensuring its isolation. In order to create a closed system to minimize blood loss, one of the sutures was used to ligate the ECA distally to the bifurcation while a microclamp was placed on the ICA. The ECA was then nicked just proximally to the ligation point and the drug delivery tubing was inserted into the nicked vessel. A suture was used to secure the tubing for the duration of drug delivery. Once the tubing was successfully placed, the mouse underwent the reperfusion phase of the tandem ipsilateral common carotid and middle cerebral artery occlusion stroke model (as described above), the clamp on the ICA was removed, and 10–25 μL drug was administered at a rate of 10 μL per minute. Following drug administration, a suture was used to ligate the ECA proximal to the nick and the tubing was removed. The mouse was then allowed to recover for the duration of the study (3 to 7 days).

#### Treatment with IL-1α

Doses administered exogenously were determined using our in vitro and in vivo dose-response experiments previously published [25, 39]. Each mouse received 0.05 μg/kg IL-1α (approximately 1 ng per 100 μL of PBS) via tail vein (IV) injection or 0.005 μg/kg via IA injection. Injections were performed on anesthetized adult mice immediately following recanalization of occluded vessels. All mice received a single dose of IL-1α on the day of surgery (acute), or on post-stroke day (PSD) 3 (delayed/subacute) and were allowed to recover up to PSD7 or PSD14 for subsequent behavioral and histologic analyses.

### Blinding and randomization

In adherence to STAIR criteria, all experiments were blinded and randomized [1]. For these exploratory studies, we used young, male mice. Future confirmatory studies will include female and aged mice. All animals were pre-assigned to groups using an online randomization generator. Additionally, other personnel were tasked with making up the IL-1α fresh on the day of use, and labeling them with the correct, blinded identifier as described above. The primary experimenter (KS) was not un-blinded until after all analyses were completed.

### Behavioral assessments

#### Eleven-point behavioral neurological score

Mice that underwent the transient tandem ipsilateral CCA/MCA occlusion model underwent behavioral assessment to assess the following behavioral metrics: level of consciousness (LOC), gaze (G), visual field (VF), sensorimotor response (SR), grip strength, and endurance/paralysis paw hang (PPH). LOC was determined prior to any disturbance of the animal’s cage and was assessed on a 0–2 severity scale with 0 being alert and active without outside stimulus, 1 being responsive to stimulus, and 2 being huddled, unresponsive, and non-grooming. Gaze was assessed by passing a visual stimulus in front of each eye in turn without disturbing the mouse’s whiskers. The subject was given a 0 score if they looked toward the stimulus, and a 1 if they failed to do so. VF was assessed by holding the mouse by the tail near a platform (on its right or left side), and if the mouse reached for the platform it received a score of 0. If it did not reach within 5 s, it was given a score of 1 for each side it failed on. SR was scored by pressing each paw in turn to elicit a reaction. A reaction was defined as vocalizing pain, retracting the paw, or jumping in response to the paw press. A lack of any of these signs resulted in a score of 1 for each paw affected. Finally, PPH was scored by a typical paw hang test. The mouse uses its front paws to hang from a rod for a period of 60 s. The mouse receives a score of 0 if it is able to hang with both paws without dropping a paw below the level of the rod for the full 60 s. A score of 1 is earned if the mouse drops either paw without falling. The time of the first “partial” paw drop is also recorded. A score of 2 is earned if the mouse falls, releasing both paws, at any time during the 60 s time period. The total scores are tallied at the conclusion of the testing to assess overall function. Other summary metrics such as “latency to first paw drop” were also used to help assess fine motor function.

#### Twenty-eight-point neurological score

Mice that underwent the filament MCAo model were scored neurologically for focal deficits with a 28-point neurological scoring system as previously reported [10]. The 28-point scale awards a score of 0–4 (0 = normal, 4 = most severely affected) on seven different characteristics by a variety of assessment methods: (i) body symmetry—assessed by observation on open bench, (ii) gait—assessed by observation on open bench, (iii) climbing—assessed by observing gripping at 45°, (iv) circling behavior—assessed by observation on open bench, (v) front limb symmetry—assessed via tail suspension, (vi) compulsory circling—assessed by allowing front limbs to be placed on bench during tail suspension, and (vii) whisker response—assessed via light touch from behind.

#### Open field behavioral assessment

Each subject was placed in its own 2 × 2 box and tracked using the EthoVision 12 software (Cincinnati, OH USA) for 5 min. Animals were assessed on the day prior to stroke surgery, and then again on PSD 1, 3, and 7. Parameters tracked include total distance traveled, average velocity, turn angle, and time spent in center zone. The center zone was defined as being all area within the box that was at least 5 in away from the walls of the box. This parameter allowed us to track anxiety as a function of how long the animal ventured into the center of the box.

### Histology

#### Morphological stains

Infarct volume was assessed using cresyl violet staining. Mounted 20-μm sections were fixed with 10% phosphate-buffered formalin. They were then stained using standard cresyl violet staining methods, mounted using DPX Mounting medium (Sigma-Aldrich, St. Louis, MO, USA), and were scanned using a HP Scanjet G4050. The scanned images were analyzed using National Institutes of Health (NIH) ImageJ software for infarct volume measurement as previously published [22]. Infarct regions were defined as regions with hypodense cresyl violet staining reflecting areas of dead or dying nuclei. Areas were calculated using the ImageJ free-hand selection tool and summated to calculate final infarct volume.

### Immunohistochemistry

Mounted, 20-μm tissue sections were fixed with ice-cold 1:1 acetone:methanol prior to incubating in blocking buffer (5% BSA in PBS with 0.1% Triton X-100) for 1 h at room temperature. The sections were then incubated overnight at 4 °C in primary antibody (in 2% BSA/0.1% Triton X-100) against PECAM (1:100, Fisher, Cat. #CBL1337) CD11b (1:200, BioRad, Cat. #MCA711G), ICAM (1:200, R&D Systems, Cat. #AF796), VEGFR2 (1:100, Abcam Cat #ab10972), and doublecortin (DCX) (1:250, Abcam Cat. #ab18723). Sections were washed and incubated with a fluorescent secondary antibody (1:1000; AlexaFluor 488 or 568, Life Technologies) for 1 h at room temperature. Alternatively, Millipore ApopTag staining kits were used as directed to stain for apoptotic cells with a terminal deoxynucleotidyl transferase dUTP nick end labeling (TUNEL) marker. Sections were washed again and then coverslipped with fluorescent mounting media containing DAPI (H-1200, Vector Labs, Burlingame, CA, USA) and images were captured using a Nikon Eclipse Ti microscope and software (Nikon). Images were analyzed for antibody-specific positive staining using ImageJ (threshold pixel intensity made similar across all images to isolate antibody-specific staining and then recorded the number of stain positive pixels). Results are from three sections per animal and the area selected was in the infarct core identified morphologically, or the peri-infarct as defined as a 500-um boundary extending from the edge of the infarct core, medial and lateral to the infarct [22].

### Cell culture

#### Primary fetal cortical neuron culture

Brains from E14-18 mouse pups were removed and placed in ice-cold HBSS solution (Corning 21-022-CV) in 100 mm petri dishes (Corning 3296). Next, the meninges, midbrain, and hippocampus were removed, leaving only cortical tissue in the dish. Dissected cortical tissue was then transferred with HBSS to a clean 15 mL conical tube (Falcon). The tissue was allowed to settle at the bottom of the tube and the HBSS was removed and replaced with 5 mL of 1 mg/mL trypsin (Sigma T9201) solution in cold HBSS. After a 20-min incubation at room temperature, the trypsin was removed and replaced with a non-trypsin neutralizing solution. After a brief incubation, to ensure deactivation of trypsin, the trypsin-neutralizing solution was removed. Dissociated cortices were then resuspended in 5 mL seeding media (Neurobasal Medium (NBM), Thermo-Fisher Scientific, UK), 5% plasma-derived serum (PDS) (First Link Ltd., UK), 1 U/mL penicillin/100 mg/mL streptomycin (P/S), 1% glutamine (Sigma-Aldrich, UK), 2% B27 supplement with antioxidants (Thermo-Fisher Scientific, UK)) and triturated. Cells were then plated at 10,000 cells per well of a poly-d-lysine coated 12-well plate. Plates were lightly agitated to ensure even distribution of primary neurons. Neurons were incubated at 37 °C and 5% CO_2_ for at least 1 week prior to use.

#### Endothelial cell culture

Brain microvascular endothelial cells (BECs) from C57BL/J6 mice maintained as cells lines ([9]; Sapatino et al. 2013) were used in this study. C57BL/6 BECs were cultured on porcine gelatin-coated tissue culture plates in Iscove’s modified Dulbecco’s medium (IMDM) containing 10% fetal bovine serum (FBS), 1% P/S, and 1% l-glutamine, and were kept at 37 °C and 5% CO_2_ and cells were grown to confluence prior to experimental use.

#### IL-1α treatment of endothelial cells

BECs were treated with IL-1α as indicated and as previously published [39]. RNA was collected 4 h following treatment (optimized from previous, unpublished studies) and purified using pureLink RNA kit (Invitrogen, Carlsbad, CA USA). RNA was then reverse transcribed using a high capacity cDNA reverse transcription kit (Applied Biosystems, Thermo Fisher Scientific) and levels of cathepsin B, and perlecan were determined using Viia7 software (Thermo Fisher Scientific, USA) and TaqMan reagents and probes specific for mouse cathepsin B and perlecan.

#### Oxygen-glucose deprivation insult and IL-1α treatment of primary neurons

After 1 week of incubation at 37 °C and 5% CO_2_, primary neuronal cell cultures, prepared from the brains of mice embryos at 14 to 16 days of gestation, as described previously [31], were subjected to 30-min oxygen-glucose deprivation (OGD) and then allowed to re-perfuse for 24 h in conditioned media containing PBS vehicle, 0.1, 1, 10, or 100 ng/mL IL-1α. These doses were chosen based on our previous studies in endothelial cells [39]. Because this study was done in neurons, we chose doses on a logarithmic scale (2 logs above and 2 logs below) in order to obtain a clear dose-response curve similar to our previous studies [39]. Cells were then labeled with Hoechst nuclear stain, fixed, visualized on a Nikon inverted microscope, and quantified for chromatin fragmentation and cellular health. Cells were classified as being healthy or unhealthy [2]. We quantified five areas per coverslip or up to 200 healthy cells with nine coverslips per treatment group.

#### NMDA insult and IL-1α treatment of primary neurons

Primary neuronal cell cultures, seeded at 1 × 10^6^ cells/mL into 24-well plates, were treated with vehicle (0.1% low endotoxin BSA), *N*-methyl-d-aspartate (NMDA) (Tocris, UK) (20 μM), IL-1α (10 ng/mL), NMDA (20 μM), and IL-1α (0.1 or 10 ng/mL) for 24 h. Each animal used for primary neuron harvest contributed to one experimental replicate so that neurons from each individual animal received all treatment groups resulting in a randomized block study design. The percentage of neuronal cell death was quantified with lactose dehydrogenase (LDH) cell death assay, normalized to percent dead of total cells and then converted to percent viability for analysis.

### Experimental design and statistical analysis

All experiments were performed in duplicated studies, and each treatment group contained at least four mice. Data are represented as mean ± standard error of the mean (SEM). Comparison between two groups was done using the Student’s *t* test. Comparison between three or more independent groups at a single time point was performed using one-way analysis of variance (ANOVA) followed by a Tukey’s post hoc analysis. NMDA toxicity analysis was performed using a randomized block design using SAS software and blocking for each animal. Comparison between three or more groups at multiple time points was performed using two-way RM ANOVA or ordinal logistic regression dependent on data type (continuous vs. ordinal). All continuous data were analyzed using GraphPad Prism Software and ordinal data was analyzed using IBM SPSS statistics 20. Significance was determined by a *p* value of < 0.05.

## Results

### IL-1α is directly protective of primary cortical neurons after OGD and NMDA toxicity in vitro

**Fig. 1:**
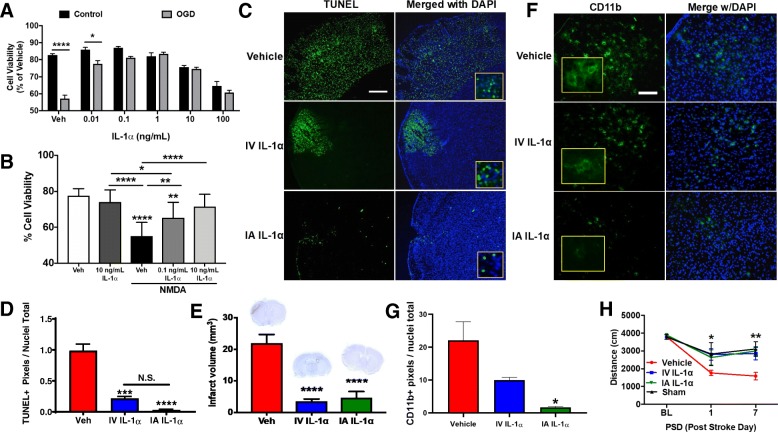
IL-1α conveys direct neuroprotection both in vitro (**a**, **b**) and in vivo (**c**–**h**) when delivered acutely**.** Primary cortical neurons under two forms of cytotoxic stress: **a** OGD or **b** 20 μM NMDA. Excess IL-1α concentrations are cytotoxic while moderate doses conveyed direct protection from oxygen-glucose deprivation (OGD) as well as NMDA-based toxicity (*n* = 9 per group). Mice treated with IA IL-1α have **c** fewer apoptotic cells in the infarct and peri-infarct regions than vehicle and IV IL-1α treated mice 3 days following stroke. **d** Quantification of TUNEL and **e** cresyl violet stains (representative images of stained sections depicted above each bar). Mice treated with IA IL-1α have reduced infarct volumes on PSD 3 compared to control mice. **c** Scale = 200 μm (*n* = 3 per group). Mice treated with IA IL-1α less microglial activation in the peri-infarct regions than vehicle or IV IL-1α treated mice on PSD 7 (**f**, **g**)**.** Representative images of CD11b (green) staining showing less overall microglial staining in the peri-infarct region of treated animals on PSD 7 compared to controls; inset showing magnified representing images (**f**) (*n* = 4 per group). Scale = 50 μm**.** Quantification of CD11b stains (**g**). IL-1α enhances functional recovery following stroke. Mice were evaluated for functional performance by using total distance traveled in an open field free movement paradigm (**h**). Mice were evaluated for a baseline measurement the day prior to stroke surgery and then evaluated for functional recovery on PSD 1 and PSD 7. Mice treated with IA or IV IL-1α show better functional outcome than control mice (*n* = 5 per group). *p < 0.05; **p < 0.01; ***p < 0.001; ****p < 0.0001. Data are the mean ± SEM

As a proof of concept, we first investigated whether IL-1α could impart protection to neurons undergoing the in vitro stroke analogue oxygen glucose deprivation (OGD) or using an in vitro model of post-stroke toxicity, exposure to NMDA. After clearly demonstrating that OGD decreased cell viability in the absence of IL-1α (control vehicle: 82.83 ± 0.79% vs. OGD vehicle 57.20 ± 2.05% viable), we found that IL-1α significantly increased the cell viability after OGD (OGD vehicle: 57.20 ± 2.05% viability vs. 1 ng/mL IL-1α: 83.45 ± 0.98%, *p* < 0.0001) (Fig. 1a). The lowest and highest concentrations of IL-1α were not as beneficial (0.01 ng/mL: 77.72 ± 1.84%, *p* < 0.005) and, in fact, the highest doses were detrimental even under normoxic conditions (100 ng/mL IL-1α under normoxia: 64.73 ± 2.43% vs. 60.68 ± 1.47% under OGD). Although the highest dose (100 ng/mL) showed toxicity, it still prevented further cellular death under OGD conditions. We also found that IL-1α significantly increased the cell viability following 20 μM NMDA insult (Fig. 1b) (20 μM NMDA: 55.17 ± 7.54% viable vs. 20 μM NMDA with 0.1 ng/mL IL-1α: 65.36 ± 8.58% viable; *p* < 0.01 and vs. 20 μM NMDA with 10 ng/mL IL-1α: 71.59 ± 6.76% viable; *p* < 0.0001) Collectively, IL-1α was directly neuroprotective in vitro in the face of different noxious conditions, supporting the idea that IL-1α, despite being an inflammatory cytokine, could have beneficial neuroprotective effects under appropriate dosing regimens.

#### Acute IL-1α administration reduces infarct volume and apoptotic cell death following stroke

We next investigated the therapeutic potential of acute IL-1α administration in experimental stroke in vivo, as well as whether it might also represent an attractive candidate for IA drug delivery using our recently developed IA drug delivery in stroke model [25]. At 3 days after stroke (PSD3), animals which received IL-1α immediately after recanalization (acute administration) showed significantly lower levels of apoptotic cell death on TUNEL staining (Fig. 1c, d) (vehicle: 14047 ± 1469 vs. IV IL-1α: 3093 ± 466.2 (*p* < 0.001) vs. IA IL-1α: 441 ± 152 (*p* < 0.0001) TUNEL positive pixels) as well as lower overall infarct volumes on cresyl violet staining (Fig. 1e) (vehicle: 21.93 ± 2.75 mm^3^ vs. IV IL-1α: 3.546 ± 0.72 mm^3^ (*p* < 0.0001) vs. IA IL-1α: 4.664 ± 0.72 mm^3^ (*p* < 0.001)). Interestingly, while IA IL-1α administration did not further lessen overall infarct volumes (measured via cresyl violet stain) compared with IV IL-1α administration (Fig. 1e), IA IL-1α further decreased apoptotic cell death compared to IV IL-1α, although this effect was not statistically significant (Fig. 1d) (IV IL-1α: 3093 ± 466.2 vs. 441.7 ± 152.2 TUNEL positive pixels *p* < 0.5).

#### Acute IL-1α administration reduces intra-parenchymal inflammatory activation after stroke

We next investigated whether IL-1α instigated widespread inflammatory activation within the brain. Unsurprisingly, we saw that stroked, vehicle-treated animals had widespread microglial (CD11b) activation (21,556 ± 3903 positive pixels) (Fig. 1f, g). However, animals receiving IV IL-1α showed decreased CD11b staining compared to control (9098 ± 1580 positive pixels; *p* > 0.02), and animals which received IA IL-1α showed even less CD11b staining compared to IV IL-1α (1952 ± 611.2 positive pixels; *p* < 0.005). It is noteworthy that CD11b staining labels both microglia and infiltrating monocytes, and therefore, is an indicator of the overall inflammatory cell load in the brain following stroke. These data suggest that the administration of low dose IL-1α could actually decrease the inflammatory response to stroke.

#### Acute IL-1α administration improves functional outcomes following stroke

Next, to determine whether neuroprotective effects of IL-1α might also correlate with functional benefit, mice underwent a battery of behavioral tests (an 11-point neurological score, and open field testing). While there were no significant differences in our compiled behavioral score (data not shown), open field testing demonstrated that both IV- and IA-treated animals traveled farther in overall distance than their vehicle control counterparts on both PSD 1 (two-way ANOVA vehicle: 1762.07 ± 157.86 cm vs. IV IL-1α: 2797.45 ± 318.49 cm; *p* < 0.05 vs. IA IL-1α: 2633.04 ± 431.55 cm; *p* < 0.5) and PSD 7 (two-way ANOVA vehicle: 1587.41 ± 209.70 cm vs. IV IL-1α: 2849.18 ± 347.31 cm; *p* < 0.005 vs. IA IL-1α: 2994.12 ± 248.34 cm; *p* < 0.005) (Fig. 1h). Additionally, we noted that animals in both IV- and IA-treated groups spent more time in the open areas of the arena rather than staying near the walls (data not shown). This suggests that these animals are both more mobile and are not exhibiting elevated anxiety compared to controls. Even more interestingly, these effects become more pronounced with IV or IA IL-1α treatment, but not in vehicle controls, with increased time after stroke. Taken together, we found that, regardless of treatment modality, acute IL-1α treatment improves functional outcomes after stroke.

#### IL-1α treatment is safe up to 10^5^ times its effective dose

**Fig. 2:**
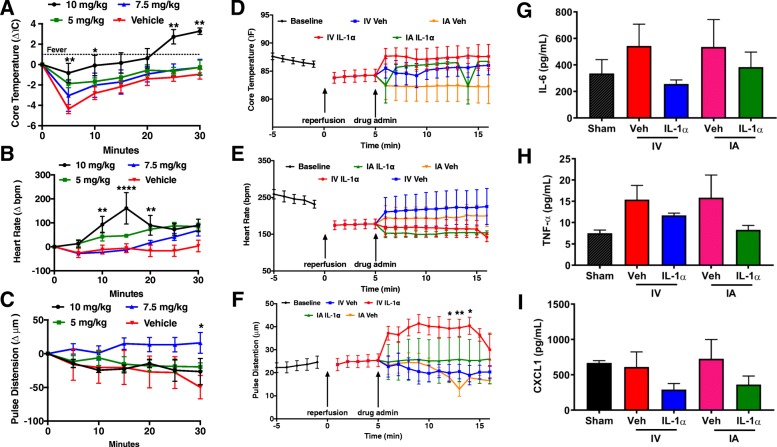
Side effects of acute IL-1α treatment following stroke vary depending on dose. **a**–**c** Larger doses of IV IL-1α after stroke can cause **a** fever, **b** elevated heart rate, and **c** elevated pulse distension (two-way ANOVA *p < 0.05; **p < 0.01; ***p < 0.001; ****p < 0.0001). **d–f** Doses of IL-1α administered IV (1 ng = 3.3 × 10^−5^ mg/kg) or IA (0.1 ng = 3.3 × 10^−6^ mg/kg) had no effect on **d** core temperature, or **e** heart rate. IV administration of IL-1α caused significant elevation of pulse distension (**f**) but this effect was lost with IA administration (two-way ANOVA *p < 0.05; **p < 0.005). Doses of IL-1α administered IV (1 ng = 3.3 × 10^−5^ mg/kg) or IA (0.1 ng = 3.3 × 10^−6^ mg/kg) had no effect of systemic (blood) levels of **g** IL-6, **h** TNF-α, and **i** CXCL1 24 h after MCAo/IL-1α treatment, compared to vehicle-treated and sham control animals. Data are the mean ± SEM (*n* = 5 per group)

It has long been established that IL-1α is an early mediator of fever and an early signaling molecule in sepsis. As this could be a potential concern for the therapeutic use of IL-1α, we wanted to identify the dose at which IL-1α might become unsafe/poorly tolerated in mice, as indicated by its ability to cause a fever. We defined a “mild” fever in mice as a 1 °C sustained increase in core body temperature and classified 2.5 or more degrees °C sustained increase in core body temperature as being a “severe” fever [33]. In animals that underwent MCAo surgery, we administered 5, 7.5, and 10 mg/kg of IL-1α via tail vein injection and monitored core body temperature by rectal probe (along with other vital statistics such as heart rate, and pulse distension (analogous to blood pressure)). None of the mice that received 5 mg/kg of IL-1α developed fever, whereas 50% of the mice that received 7.5 mg/kg developed fever with at least one of them developing severe fever. Finally, 75% of the mice receiving 10 mg/kg developed fever, all of which was sustained severe fever (Fig. 2a)**.** Only the 10 mg/kg dose caused a rapid and sustained elevation of core temperature following injection (5 min *p* < 0.005, 10 min *p* < 0.05, 25 min *p* < 0.01, and 30 min *p* > 0.001) and transiently elevated heart rate compared to vehicle (10 min *p* < 0.01, 15 min *p* < 0.0001, 20 min *p* < 0.01, and losing significance at 25 (*p* < 0.1) and 30 (*p* < 0.5) minutes) (Fig. 2b). The 7.5 mg/kg dose slowly elevated pulse distension (30 min *p* < 0.05) compared to vehicle (Fig. 2c). This suggests that the animals tolerate IL-1α up to 10^5^-fold our chosen IV post-stroke dose.

#### Intra-arterial IL-1α treatment prevents transient hemodynamic changes

Having shown that IL-1α is non-lethal and presents minimal side effects up to 10^5^-fold our chosen IV dose and having shown the added histological benefit of IA IL-1α (Fig. 1), we compared the routes of administration for potential differences in effects on core body temperature (Fig. 2d), heart rate (Fig. 2e), and pulse distension (Fig. 2f). The route of administration (IV vs. IA) did not significantly affect either core temperature (Fig. 2d) or heart rate (Fig. 2e) at any time following drug administration. Interestingly, IV, but not IA, IL-1α caused a significant increase in pulse distension (Fig. 2f) at 12 (*p < 0.05), 13 (**p < 0.005), and 14 (*p < 0.05) minutes following reperfusion (7, 8, and 9 min following drug injection respectively). These findings suggest that targeted IA treatment with IL-1α could both improve post-stroke outcomes while minimizing/eliminating hemodynamic side effects.

#### IL-1α treatment does not elevate systemic pro-inflammatory mediators in serum

In order to determine and compare the potential inflammatory systemic effects of IA and IV IL-1α delivery, we evaluated levels of known pro-inflammatory cytokines in the serum of mice recovering from stroke with or without IL-1α treatment. To do this, animals were stroked and acutely treated with IV or IA IL-1α. We then collected serum from the same animals 24 h post-stroke/treatment and then again upon sacrifice at PSD 7 to evaluate if there were effects on pro-inflammatory cytokine levels in the serum in response to not only the stroke but also in response to IL-1α injection. We found that while neither IA nor IV IL-1α administration significantly elevated systemic IL-1β, IL-6, or CXCL-1 (at PSD 1, IL-1β: *p* < 0.7; IL-6: *p* < 0.95; and CXCL-1: *p* < 0.7; *n* = 4), they, perhaps surprisingly, decreased (trend) their levels as compared to their respective vehicle controls (Fig. 2g–i). Collectively, this and our previous results (Fig. 2a–f) suggest that post-stroke administration of both IV and IA IL-α is safe.

#### Delayed/subacute IL-1α-treated animals have improved functional outcomes

We next investigated whether delayed/subacute IL-1α treatment enhances functional benefit when given 3 days following stroke. These studies were performed in parallel in both the tandem transient CCA/MCA occlusion model, and the filament MCAo model. In both stroke models, both treatment groups showed similar functional deficit in their total scores on the days following stroke surgery as expected (filament MCAo 28-point score: vehicle: 10.57 ± 1.39 vs. IL-1α: 10.60 ± 1.24 points, tandem CCA/MCA model not shown). In the filament model, the mice treated with IL-1α exhibited consistently declining scores (i.e., improved function, ordinal logistic regression ***p < 0.005) compared to vehicle. Similar results were noted with the tandem CCA/MCA model (data not shown).

#### Delayed/subacute IL-1α-treated animals have more vascular density in the peri-infarct region

**Fig. 3:**
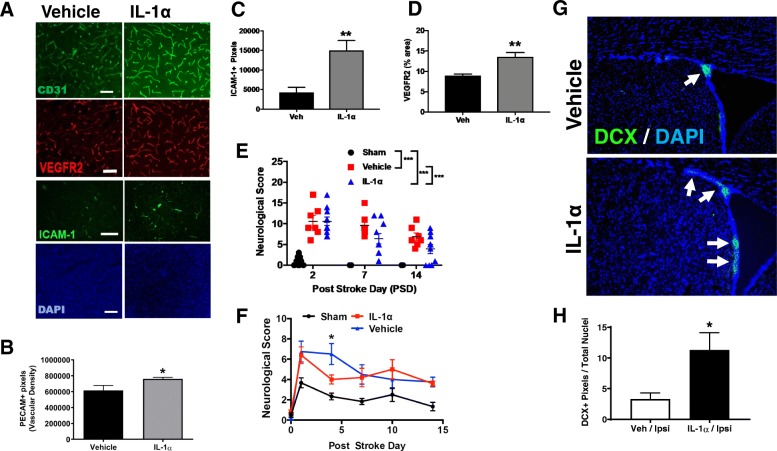
Delayed/subacute treatment with IV IL-1α enhances post-stroke recovery and repair. Delayed/subacute IL-1α treatment increases expression of markers of vascular activation (**a–d**) and early neurogenesis (**g**, **h**). Representative images of stains for CD31 (PECAM), ICAM-1, and VEGFR2 (**a**). Quantification of **b** PECAM, **c** ICAM-1, and **d** VEGFR2 stains. These stains show more vascularization and more EC activation 14 days following stroke. Delayed, single dose (**e**) and subacute doses (**f**) of IL-1α imparts functional benefit after stroke. Graphs showing increased functional recovery on 28-point neuroscore at 7- and 14-days following stroke in the filament MCAo model. Representative images of brains from stroked mice stained (**g**) for doublecortin (DCX) at the subventricular zone (SVZ) 14 days following stroke. Quantification of DCX stains (**h**) show more DCX-positive staining at the SVZ with IL-1α treatment compared to vehicle-treated control animals. Student’s *t* test *p < 0.05; **p < 0.01. One-way ANOVA **p < 0.01; ***p < 0.001. Two-way RM ANOVA *p < 0.05. Scale = 100 μm. Data are the mean ± SEM (*n* = 5 per group)

Because of our previous work demonstrating that IL-1α could enhance brain angiogenesis in vitro [39], we next examined whether delayed/subacute IL-1α treatment might affect post-stoke angiorepair. We first examined overall peri-infarct vascular density using PECAM-1 (CD31) and found that IL-1α-treated animals had increased overall vascular density in the peri-infarct region (Fig. 3a–d) (vehicle: 615106 ± 62,943 positive pixels vs. IL-1α: 761564 ± 18,901 positive pixels Student’s *t* test *p* < 0.1).

#### Delayed/subacute IL-1α-treated animals have more activated endothelial cells in the peri-infarct region

Next, we investigated whether our overall histological findings as well as the observed functional benefit could correlate with an augmented angiogenic response. Tissue sections were stained for ICAM-1 and VEGFR2, two known markers of endothelial cell activation. We found that animals treated with IL-1α had significantly more vascular ICAM-1 staining (Fig. 3a, c) (vehicle: 4317 ± 1247 positive pixels vs. IL-1α: 15,000 ± 2551 positive pixels Student’s *t* test *p* < 0.01) and VEGFR2 (Fig. 3a, d) (vehicle: 8.94 ± 0.45% area vs. IL-1α: 13.59 ± 1.03% Student’s *t* test *p* < 0.005) positive staining in the peri-infarct region than did animals receiving vehicle treatment. In contrast, IL-1α had no effect on microglial activation (mean activation (Iba-1 positive) score vehicle: 1.64 ± 0.08 vs. IL-1α: 1.54 ± 0.09 Student’s *t* test *p* < 0.8) and on astrocyte activation (mean GFAP positive percentage area vehicle: 44.58 ± 7.10 vs. IL-1α: 59.15 ± 3.89 Student’s *t* test *p* < 0.1, data not shown).

#### Delayed/subacute IL-1α-treated animals show greater expression of doublecortin at the subventricular zone

We next investigated whether delayed/subacute IL-1α could also impact post-stroke neurogenesis, an additional reparative process. To investigate this, we immunostained brains from the above experiments (sacrificed at PSD 14) for doublecortin (DCX), a marker of immature neuroblasts. We found that animals receiving delayed/subacute IL-1α had significantly more DCX-positive staining at the subventricular zone (SVZ) (Fig. 3g, h) (vehicle: 9470 ± 2742 positive pixels vs. IL-1α: 36644 ± 11,553 positive pixels Student’s *t* test *p* < 0.05).

#### Perlecan plays an important role in IL-1α-mediated neuroprotection after stroke

**Fig. 4:**
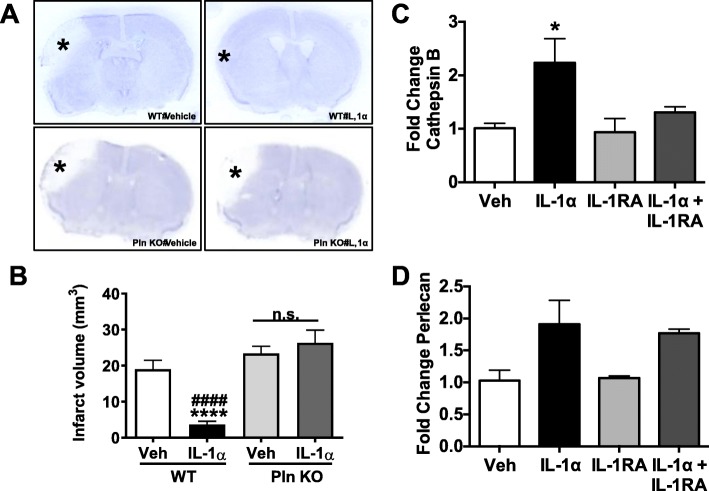
IL-1α acts through proteolytic processing of perlecan. Mice lacking the LG3 fragment of perlecan do not sustain the same protection following IV IL-1α treatment showing larger infarct volumes overall than WT controls on PSD 3 (**a**). Quantification of infarct volumes obtained from cresyl violet stains (**b**) one-way ANOVA ####*p* < 0.0001 WT IL-1α vs. pln KO IL-1α; ****p < 0.0001 WT PBS vs. WT IL-1α (*n* = 7 per group). IL-1α treatment increases mRNA expression of cathepsin B and perlecan in vitro. Mouse brain endothelial cells treated with 1 ng/mL IL-1α for 4 h express more cathepsin B (**c**) and perlecan (**d**) mRNA. This effect is abolished for the production of cathepsin B but not perlecan upon treatment with IL-1RA. One-way ANOVA *p < 0.05 IL-1α vs. Veh conditions. Data are the mean ± SEM

Our previous work suggested that elements of the extracellular matrix, such as the heparan sulfate proteoglycan perlecan, are proteolyzed into smaller protein fragments [22, 38] after stroke, and that this process could partially be driven by IL-1α [37]. Additionally, we determined that one of these proteolytic fragments, perlecan LG3, is neuroprotective following OGD [38]. To determine whether perlecan is required for the acute neuroprotective effects of IL-1α after stroke, we used a perlecan hypomorph (pln KO) mouse that expresses 10% of normal total perlecan levels (and hence 10% of normal total perlecan LG3; complete perlecan knockout mice are embryonic lethal). Importantly, while WT mice exhibited neuroprotection on PSD3 following acute post-stroke IV IL-1α treatment (infarct volume WT vehicle: 18.99 ± 2.50 mm^3^ vs. WT IL-1α: 3.65 ± 0.8921 mm^3^ one-way ANOVA *p* < 0.001), IL-1α was not neuroprotective in pln KO mice (infarct volume pln KO vehicle: 23.38 ± 1.99 mm^3^ vs. pln KO IL-1α: 26.36 ± 3.50 mm^3^ *p* < 0.5) (Fig. 4a, b).

#### IL-1α treatment increases mRNA expression of perlecan and cathepsin B in vitro

Finally, to investigate whether IL-1α could be angiogenic through fragments of perlecan (LG3 stimulates brain endothelial cell proliferation in vitro, [9]), we examined whether IL-1α treatment in vitro could affect brain endothelial cell transcription of perlecan and cathepsin-B, a protease that is known to generate LG3 from perlecan [37, 38], as detailed in our previous work [39]. We also examined whether any such effect might be IL-1 receptor type 1 (IL-1R1) mediated by using the IL-1R1 antagonist IL-1 receptor antagonist (IL-1RA). Four hours after treatment, we found that IL-1α treatment showed significant increases in cathepsin B transcription (Fig. 4c) (vehicle: 1.01 ± 0.093-fold increase vs. IL-1α: 2.24 ± 0.45-fold increase, *p* < 0.05) and near-significant increases in perlecan transcription (Fig. 4d) (vehicle: 1.03 ± 0.16-fold increase vs. IL-1α: 1.909 ± 0.38-fold increase, *p* < 0.1). Furthermore, we found that the increase in cathepsin B, but not in perlecan, was largely abolished in the presence of IL-1RA (Fig. 4c, d).

## Discussion

In the present study, we evaluated whether the pro-inflammatory cytokine IL-1α could be therapeutic in two distinct experimental ischemic stroke models. IL-1α is one of the first cytokines upregulated after stroke [23], and we recently demonstrated that IL-1α treatment of brain endothelial cells showed pro-angiogenic effects in vitro [39]*.* Thus, IL-1α is present early in stroke pathogenesis, modulates brain endothelial cell angiogenesis, and could, therefore, be a prominent component of the brain’s response to ischemic injury. We found that IL-1α was neuroprotective to primary cortical neurons under both OGD and NMDA exposure at specific doses. The highest doses and lower doses were not significantly protective and, in the case of the highest dose (100 ng/mL), IL-1α was neurotoxic. This indicated to us that it was essential to determine the proper in vivo dosing of IL-1α that might impart benefit without significant safety risk.

In order to confirm that the chosen dose of 0.05 μg/kg (based on our published in vitro studies [38];) was safe, we attempted to determine the LD_50_ of IL-1α in mice. Interestingly, our highest dose, 10 mg/kg, which was 10^5^-fold greater than the established dose, was not lethal. This shifted our focus toward determining whether any of the doses tested resulted in negative side effects. As IL-1α is a known mediator of fever, we chose fever as our symptom for decreased tolerance [5]. As described above, we found that 7.5 mg/kg produced fever in 50% of the mice out to at least 30 min following recanalization. While none of these mice died during or after the injections, these results clearly show that injected IL-1α was both active and could, at high enough doses, produce severe fever as well as other hemodynamic changes such as changes in blood pressure and heart rate. These results demonstrate that there is a large safe/well-tolerated potential dosing range for IL-1α.

After determining that our established dose was safe, we demonstrated that acute, single-dose IL-1α treatment in stroked mice is neuroprotective. However, when given IV, IL-1α results in transient, mild hemodynamic changes in pulse distension (analogous to blood pressure). Fortunately, post-stroke IA delivery allowed us to both administer less IL-1α, and to deliver IL-1α in a stroke-targeted fashion, collectively reducing hemodynamic side effects. Additionally, since it is known that IL-1α is readily transported across the BBB [4], coupled with the well-documented post-stroke disruption of the BBB, we are confident that at least some of the administered IL-1α was taken up into the brain parenchyma.

Acute post-stroke IV or IA IL-1α administration resulted in comparable significant reductions in ischemic infarct volume, fewer apoptotic cells, improved functional recovery, and decreased neuroinflammatory activation, the latter with IA treatment being more effective than IV treatment. There are several potential reasons for this differential reduction in neuroinflammation. We administered a much smaller IA versus IV IL-1α dose as dosing of drugs to the central nervous system (CNS) is historically far lower than similar effective IV doses of the same drug; examples of such dose minimization include IA chemotherapy for retinoblastoma [49], as well as IA thrombolysis with tissue plasminogen activator (tPA) for ischemic stroke [19]. In such cases, the IA doses typically are 1/10 or less of the systemically administered dose. The smaller IA dose may simply have resulted in less induction of inflammation than the larger IV dose of this inflammatory cytokine. Furthermore, IL-1α could be working locally through another mechanism of neuroprotection thereby reducing the inflammatory response secondary to smaller overall injury.

Taken together, we were able to use IL-1α in combination with our recently developed IA drug delivery and stroke model as a proof of concept for giving potentially life-saving drugs with a safer drug delivery mechanism. Endovascular thrombectomy gives clinicians a great opportunity to deliver drugs in a targeted fashion immediately following vessel recanalization [25]. Our stroke model and combined IA drug delivery method model’s clinical large vessel occlusion and this targeted drug delivery very closely [22, 25]. Our current and previous results suggest that stroke therapeutics that have been previously discarded on the basis of producing side effects, or minimal efficacy upon peripheral administration might merit re-examination as IA therapy [26, 27].

As our previous work suggested that IL-1α could promote brain angiogenesis in vitro [39], we also investigated the potential reparative effects of IL-1α in the context of stroke in vivo. In an attempt to separate the neuroprotective effects of IL-1α from its neuroreparative effects, in these experiments we delayed IL-1α administration until PSD3, a time point at which the ischemic infarct is maximally evolved in our stroke model [22]. Furthermore, such delayed treatment was IV administered, as delayed IA administration would have involved a second surgery to again isolate the carotid artery circulation, etc. Our previous research demonstrated that overall expression of IL-1α in the brain remained elevated up to a week after stroke [39]. However, others have more recently discovered that IL-1α is elevated out to at least 7 weeks following ischemic injury [13]. These observations suggest that endogenous IL-1α could play a chronic role in the brain’s response to injury that might be augmented by delayed exogenous administration as done in the current study. Indeed, we also saw that delayed/subacute IL-1α-treated mice showed less overall damage, more overall vascularization and brain endothelial cell activation within the peri-infarct area, more DCX-positive cells in the SVZ, and functional improvement. Importantly, the delayed IL-1α treatment paradigm was validated in two different stroke models in two different labs, meeting an important criterion for the STAIR recommendations for the testing on experimental stroke treatments [1]. An important future direction for this study would be to investigate the role of stem cells derived from other brain areas, as there is increasing evidence showing the limited migration and neuron-generating abilities of SVZ-derived stem cells [20, 32]. Regionally derived stem cells such as reactive astrocytes [17, 42], oligodendrocyte precursor cells [21], radial glia-like cells [6], and reactive pericytes [29, 30] may also be functioning as endogenous stem cells that differentiate into neurons. Therefore, although we observed neurogenesis in the SVZ, other brain region-derived stem cells and their roles in the post-stroke brain, especially following IL1α treatment needs to be explored.

It has been reported that polymorphisms in the IL1A gene are linked to higher incidences of vascular malformation and possibly ischemic stroke [44, 45]. Based on our findings, it seems likely that these polymorphisms (− 889 and + 4845 bp positions from the transcription start site) lead to reduced IL-1a activity via release of a less active form, which would in turn, impair blood-brain barrier integrity and function. This is consistent with our results suggesting that IL-1α used in this study has neuroprotective effects.

Our results demonstrating increased peri-infarct vascularization and vascular activation are consistent with our previous in vitro observations that demonstrated that IL-1α stimulates brain endothelial cell activation, proliferation, migration, and capillary morphogenesis in vitro [39]. Furthermore, our doublecortin result, while suggestive of increased post-IL-1α treatment-mediated neurogenesis, requires further investigation to determine whether such an increase in neuroblasts might translate into more functioning neurons in the site of injury. Additionally, more studies (potentially in animals with impaired post-stroke angiogenesis or neurogenesis capabilities) are necessary to determine whether both of these observations are merely correlative with- or also contribute to IL-1α’s therapeutic benefits. Furthermore, since C57Bl/J6 mice have a considerable degree of variability in their vascular architecture [43], between individual mice, it would be insightful to conduct these experiments in mice that exhibit somewhat more consistent vasculature, such as the CB17 strain or SCID mice.

Finally, our previous studies demonstrating that IL-1α could drive the production of the neuroprotective and angiogenic and neurogenic LG3 perlecan protein fragment from brain endothelial cells in vitro led us to hypothesize that IL-1α could be neuroprotective and neuroreparative in vivo via perlecan LG3 [37, 38]. To test this hypothesis, we used perlecan hypomorph (pln KO, expressing approximately 10% of normal physiological levels of perlecan) mice in post-stroke IL1α administration experiments. This was done because complete perlecan knockout animals were embryonically lethal, making the hypomorph animals were the only viable alternative. In support of the hypothesis, we demonstrated that IL-1α lost its neuroprotective effects in stroked pln KO mice, strongly suggesting that perlecan, and potentially its LG3 fragment, are both required and an important component for IL-1α’s neuroprotective activity. Additional in vitro studies with brain endothelial cells further demonstrated that IL-1α could also drive the production of both perlecan and the LG3-generating protease cathepsin B, further supporting the potential involvement of perlecan LG3 in IL-1α’s therapeutic effects. Interestingly, our IL-1RA results suggest that IL-1α exerts IL-1R receptor dependent and independent effects on cathepsin-B and perlecan transcription in brain endothelial cells, respectively. While the potential involvement of IL-1R in IL-1α’s therapeutic effects remains to be confirmed in vivo, our in vitro results suggest a complex mechanism of action that could shed light on why IL-RA stroke therapy has met with mixed success. Furthermore, we expect to confirm in future studies that blockade of cathepsin B (which would decrease LG3 levels, [37, 38]) would mitigate or prevent IL-1α neuroprotection in vitro, and that post-stroke administration of IL-1α also increases brain LG3 levels in wild-type mice in vivo which would further support the importance of LG3 in the therapeutic mechanism of action of IL-1α.

## Conclusions

Taken together, our results show that IL-1α significantly increases neuroprotection when administered acutely, and enhances peri-infarct brain vascular density, and potentially neurogenesis, with delayed administration. We have also established the safe and effective dosing range and routes of IL-1α administration in mice and, in so doing, have identified an attractive target for future drug discovery studies. Finally, we have provided evidence of the potential therapeutic mechanism of action of IL-1α (perlecan LG3), which will be explored further in subsequent studies.

## Data Availability

The datasets used and/or analyzed during the current study are available from the corresponding author upon reasonable request.

## Abbreviations

ANOVA: Analysis of variance
BBB: Blood-brain barrier
BSA: Bovine serum albumin
CBF: Cerebral blood flow
CCA: Common carotid artery
CNS: Central nervous system
DCX: Doublecortin
ECA: External carotid artery
FBS: Fetal bovine serum
G: Gaze
IA: Intra-arterial
ICA: Internal carotid artery
IL-1: Interleukin-1
IL-1α: Interleukin-1 alpha isoform
IL-1β: Interleukin-1 beta isoform
IMDM: Iscove’s modified Dulbecco’s medium
IV: Intravenous
KO: Knockout
LDH: Lactose dehydrogenase
LOC: Level of consciousness
MCA: Middle cerebral artery
MCAo: Middle cerebral artery occlusion
NBM: Neurobasal medium
NIH: National Institutes of Health
NMDA: N-methyl-d-aspartate
OGD: Oxygen glucose deprivation
P/S: Penicillin/streptomycin
PDM: Plasma-derived serum
PLN: Perlecan
PPH: Paralysis Paw Hang
PSD: Post-stroke day
SEM: Standard error of the mean
SR: Sensorimotor response
SVZ: Subventricular zone
t-PA: Tissue plasminogen activator
TUNEL: Terminal deoxynucleotidyl transferase dUTP nick end labeling
UK: United Kingdom
USA: United States of America
VF: Visual field
